# Investigating Polyhydroxyalkanoate Synthesis for Insights into Drug Resistance in *Xanthomonas oryzae* pv. *oryzae*

**DOI:** 10.3390/ijms26041601

**Published:** 2025-02-13

**Authors:** Qingbiao Xie, Guangshu Lao, Yukai Fang, Xue Gao, Zheng Tan, Weiguo Miao, Pengfei Jin

**Affiliations:** Key Laboratory of Green Prevention and Control of Tropical Plant Diseases and Pests, Ministry of Education, School of Tropical Agriculture and Forestry, Hainan University, Haikou 570228, China; xieqingbiao@hainanu.edu.cn (Q.X.);

**Keywords:** polyhydroxyalkane, *Xanthomonas oryzae* pv. *oryzae*, *phaC/phaZ*, HN-2 n-butanol extract, drug resistance

## Abstract

Polyhydroxyalkanoates (PHAs), synthesized by *Xanthomonas* to endure adverse conditions, are primarily regulated by the critical genes *phaC* and *phaZ*. Poly-3-hydroxybutyrate (PHB), a common polyhydroxyalkanoate (PHA), has been implicated in metabolism, pathogenicity, and various physiological processes in *Xanthomonas oryzae* pv. *oryzae* (*Xoo*). In this study, we investigated the effects of HN-2 using n-butanol extract (HN-2 n-butanol extract) derived from *Bacillus velezensis* on *Xoo*. The results showed that HN-2 n-butanol extract could induce PHB accumulation in *Xoo*, potentially via surfactin. Moreover, examination of drug resistance, pathogenicity, and morphological characteristics of *Xoo* revealed PHB played a significant role in the drug resistance, pathogenicity, membrane integrity, and growth rate of *Xoo* strains following the deletion of *phaZ* and *phaC*. The ∆*phaZ* strain was the most significant, with a growth rate reduced to 58.19% of the *PXO99^A^* at 36 h and an inhibition zone 57.46% larger than that of *PXO99^A^* by HN-2 n-butanol extract. Transmission electron microscopy further revealed blank spots in *Xoo* after treatment, with the fewest spots observed in ∆*phaZ*, indicating its impaired ability to repair and maintain membrane integrity. These findings offer valuable insights that could serve as a foundation for elucidating the mechanisms of drug resistance and future research on preventing *Xoo*-induced diseases.

## 1. Introduction

*Xanthomonas oryzae* pv. *oryzae* (*Xoo*) is a common pathogen that primarily causes rice bacterial blight [1,2]. Bacterial leaf blight (BLB) is a particularly significant disease affecting rice in all rice-growing regions globally, which impacts rice plants at any growth stage, leading to substantial yield losses worldwide [3,4,5]. Traditionally, BLB has been controlled using chemical bactericides, such as bismerthiazol, zinc thiazole, and thiodiazole copper. However, the over-reliance on chemical control methods has contributed to the emergence of drug-resistant pathogen strains and poses risks to environmental safety [6,7,8]. Therefore, the biological control offers a more environmentally friendly and promising alternative for pathogen management. *Bacillus* species, in particular, are regarded as important biological control agents, producing a wide range of biologically active secondary metabolites that can inhibit the growth of plant pathogens and deleterious rhizospheric microorganisms [9,10,11,12,13].

Polyhydroxyalkanoates (PHAs) are natural polyesters containing various hydroxyalkanoates (HAs) synthesized by microorganisms [14]. These compounds have garnered attention as potential raw materials for environmentally friendly products, such as alternatives to conventional petroleum-based plastics and elastomers [15,16,17]. In bacteria, PHAs accumulate as discrete granules through five main biosynthetic pathways: the glycolytic pathway, the pentose phosphate pathway, the Krebs cycle, and the pathway for amino acid and fatty acid biosynthesis and degradation [18,19,20]. These synthetic pathways are directly or indirectly linked to many central metabolic processes. When the nutrient supplies are imbalanced, the bacteria can polymerize the soluble intermediates into low-soluble molecules, such as PHAs, without compromising the overall health. This mechanism allows bacteria to store excess nutrients and maintain internal conditions, preventing detrimental changes during nutrient accumulation [19]. Furthermore, PHAs serve as energy storage compounds that help bacteria withstand adverse conditions [14,21,22]. Among PHAs, poly-3-hydroxybutyrate (PHB) is the most extensively studied. PHB synthesis involves three reaction steps: first, β-ketothiolase (phaA) catalyzes the condensation of two acetyl-CoA molecules to form acetoacetyl-CoA. In the second step, acetoacetyl-CoA is reduced to (R)-3-hydroxybutyryl-CoA [(R)-3-HB-CoA] by acetyl-CoA reductase (phaB). Finally, PHA polymerase (phaC) catalyzes the polymerization of (R)-3-HB-CoA into a growing PHB chain [23,24]. The bacteria’s ability to accumulate PHB is also considered a distinguishing feature for classification [25]. When carbon sources are abundant but other nutrients, such as nitrogen, are limited, bacteria store excess carbon as PHAs through PHA polymerase (phaC). In known PHA biosynthetic pathways, acetyl-CoA serves as a key intermediate produced in the glycolytic pathway and is an essential precursor for synthesizing various short-chain and medium-chain PHAs.

Bacteria could degrade PHAs under starvation conditions, releasing R-hydroxyalkanoic acid via PHA depolymerase (phaZ), which then serves as a carbon and energy source [26,27]. It has also been suggested that PHB contributes to bacterial resistance against adverse conditions. For example, the presence of PHB granules enhances bacterial survival under hypertonic conditions by partially repairing and stabilizing cell membranes during plasmolysis [28]. In *Xoo*, a putative cytoplasmic regulator of PHB synthesis, phaR, has been shown to influence multiple bacterial characteristics, including EPS production, growth rate, motility, and virulence in plants [29]. In our previous studies, we identified that PHB in *Xoo* is closely associated with its metabolism, pathogenicity, and other physiological processes. However, the relationship between PHA/PHB synthesis and the drug resistance of *Xoo* remains unclear.

In this study, we discovered that HN-2 n-butanol extract derived from the fermentation broth of *Bacillus velezensis* HN-2, a strain previously isolated from soil, exhibits significant antibacterial effects against *Xoo* [30,31,32]. The control efficacy of the HN-2 n-butanol extract was markedly enhanced in the Δ*phaC*, Δ*phaZ*, and Δ*phaC*/Δ*phaZ* strains compared to the wild-type *PXO99^A^*. Furthermore, the HN-2 n-butanol extract was found to induce PHB production in *Xoo*. Based on these findings, our research focused on investigating the relationship between PHB biosynthesis and the drug resistance of *Xoo*, as well as exploring the effects of the HN-2 n-butanol extract on *Xoo*. These insights could provide a new theoretical basis and practical approaches for developing future strategies to prevent and control *Xoo*-induced diseases.

## 2. Results

### 2.1. Characteristics of phaC and phaZ Gene Involved in Poly-3-Hydroxybutyrate (PHB) Synthesis in PXO99^A^

**Figure 1:**
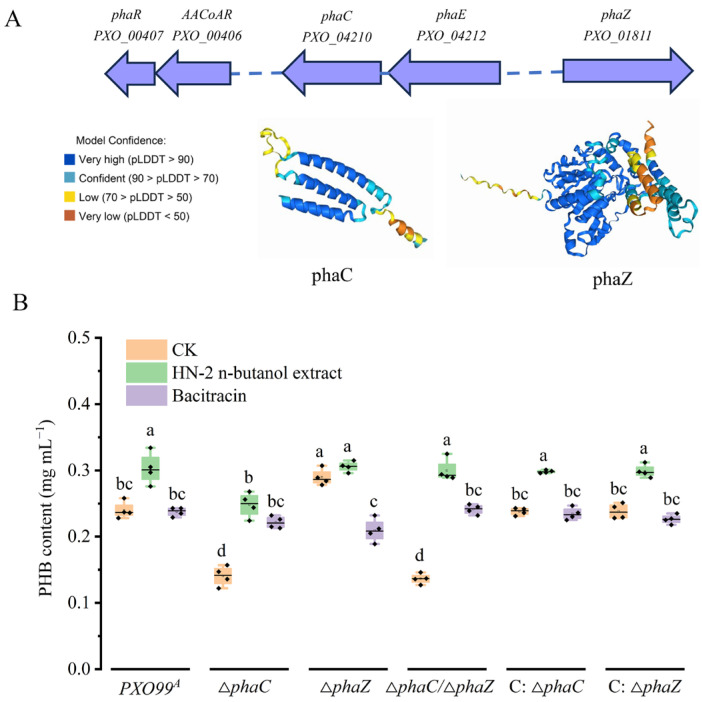
Characteristics of PHA metabolism-related genes. The PHA metabolism-related genes cluster distribution in *PXO99^A^* genome and phaC and phaZ protein structure predicted by AlphaFold2 (**A**). Content analysis of PHB from *phaC/phaZ* deficiency strains (**B**). The data are shown as the means with SD (±SD) with two-way ANOVA followed by Tukey’s multiple mean comparisons test method, the letters represent significance, *p* < 0.05.

Based on the genomic sequence annotations of *Xanthomonas oryzae* pv. *oryzae* (*Xoo*) strain *PXO99^A^* [33], we analyzed related genes involved in polyhydroxyalkanoates (PHAs) metabolism. The results show that there are five genes that were identified, including the gene encoding acetoacetyl-CoA reductase (*AACoAR*, *PXO_00406*), the PHA synthesis repressor (*phaR*, *PXO_00407*) gene, the poly (R)-hydroxyalkanoic acid synthase subunit PhaC (*phaC*, *PXO_04210*) gene, the PHA synthase subunit (*phaE*, *PXO_04212*) gene, and poly-3-hydroxybutyrate (PHB) depolymerase (*phaZ*, *PXO_01811*) gene. In *Xoo*, Genes involved in PHA synthesis form gene clusters on its genomes; *PhaR* and *AACoAR* were adjacent (Figure 1A); *PhaR* regulated the *AACoAR*, *phaC,* and *phaE* expression involved in PHA metabolism, especially affecting the expression of *phaZ*, which is responsible for the degradation of PHB granule, perhaps the most common type of PHAs [29]. Additionally, PHAs are biodegradable polyesters synthesized by most bacterial genera and some archaea as intracellular carbon and energy storage materials under unbalanced carbon or nitrogen sources and nutrient-limited conditions [27]. However, there are few reports on the role of PHAs in bacteria under drug stress. To further elucidate the PHB biosynthesis and degradation in *Xoo*, we constructed the 3-dimensional structure of phaC (pTM = 0.7) and phaZ (pTM = 0.87) via AlphaFold2 (https://golgi.sandbox.google.com/) (Accessed on 10 October 2024) (Figure 1A).

Subsequently, PHB biosynthesis was analyzed in *PXO99^A^* and its derived mutants, as we previously reported [30]. The HN-2 n-butanol extract, predominantly containing surfactin [32], was utilized for treatment and analysis. The results indicated that the HN-2 n-butanol extract promoted PHB production or accumulation in all tested strains. Notably, the PHB content in the ∆*phaC* strain increased with either the HN-2 n-butanol extract or commercial bacitracin compared to the untreated ∆*phaC*. However, the PHB levels remained lower than those in wild-type *PXO99^A^* under HN-2 n-butanol extract treatment, suggesting the presence of an alternative PHB biosynthesis pathway in *Xoo*. Additionally, the distinct effects of HN-2 n-butanol extract and commercial bacitracin on PHB levels in the ∆*phaZ* suggested that HN-2 n-butanol extract may target a different pathway in *Xoo*, highlighting its potential as a commercial agent for preventing *Xoo*-induced disease (Figure 1B). Collectively, these findings imply that phaC and phaZ are primary genes involved in PHA metabolism and may contribute to against HN-2 n-butanol extract in *Xoo*.

### 2.2. Loss of phaC/phaZ Reduced Xoo Drug Resistance to HN-2 n-Butanol Extract

**Figure 2:**
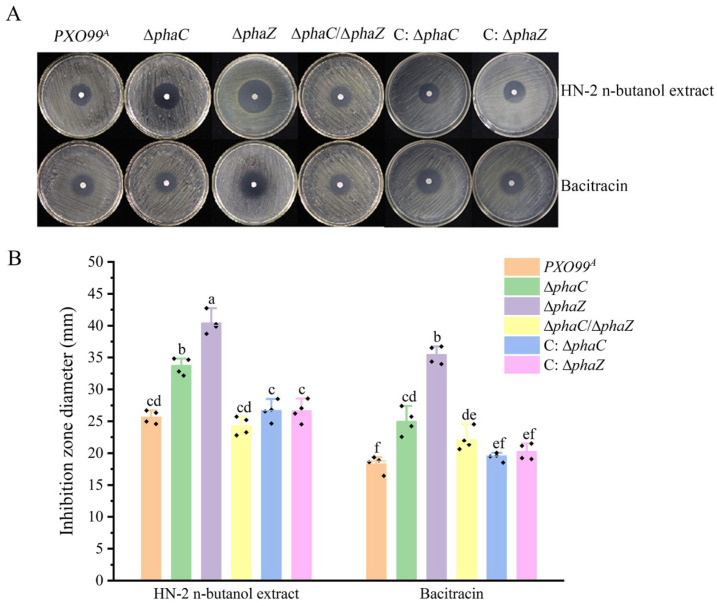
Investigation of drug-resistance of *Xoo* to HN-2 n-butanol extract. Disk diffusion assays were conducted to evaluate the sensitivity of the wild-type strain *PXO99^A^*, ∆*phaC*, ∆*phaZ*, ∆*phaC*/∆*phaZ*, C: ∆*phaC*, and C: ∆*phaZ* to the HN-2 n-butanol extract, using 6-mm diameter paper disks. Bacitracin was used as a control for comparison (**A**). The diameters of the inhibition zones for the HN-2 n-butanol extract were measured and statistically analyzed (**B**). The data are shown as the means with SD (±SD) with two-way ANOVA followed by Tukey’s multiple mean comparisons test method, the letters represent significance, *p* < 0.05.

Deletion of the *phaC* gene resulted in a reduction in PHB content, while deletion of the *phaZ* gene led to an increase in PHB content compared to the wild-type *Xoo PXO99^A^*. Furthermore, the PHB content in the ∆*phaC* strain increased following treatment with the HN-2 n-butanol extract, compared to the untreated ∆*phaC* strain (Figure 1B). However, whether PHB content is associated with the drug resistance of *Xoo* remains unknown. To investigate drug resistance of *Xoo* against HN-2 n-butanol extract, bacitracin was used as a positive control. The results showed that the inhibitory effect of HN-2 n-butanol extract and bacitracin on ∆*phaZ* was the most significant, with inhibition zone diameters of 40.39 ± 1.70 mm and 35.40 ± 1.43 mm, respectively. Additionally, the inhibitory effects of the HN-2 n-butanol extract and bacitracin on the ∆*phaC* were also notable, with inhibition zone diameters of 33.62 ± 1.33 mm and 24.98 ± 2.06 mm. However, there were no significant differences in the inhibitory effects on the wild-type *PXO99^A^*, ∆*phaC*/∆*phaZ*, and the complementary strains (C: ∆*phaC*, C: ∆*phaZ*) under HN-2 n-butanol extract treatment (Figure 2). This is likely due to the activation of alternative compensatory pathways for PHB synthesis in the ∆*phaC*/∆*phaZ*, which maintains in vivo PHB balance.

To further analyze the changes in drug resistance of the *Xoo* strains (*PXO99^A^*, ∆*phaC*, ∆*phaZ*, ∆*phaC*/∆*phaZ*) to the HN-2 n-butanol extract and bacitracin, the 50% minimum inhibitory concentration (MIC_50_) was determined. The results were presented in Table 1. The MIC_50_ values of the HN-2 n-butanol extract for ∆*phaC* and ∆*phaZ* were 0.282 μg mL^−1^ and 0.213 μg mL^−1^, respectively, significantly lower than the value for *PXO99^A^* (0.450 μg mL^−1^). In contrast, the MIC_50_ values of bacitracin for *PXO99^A^*, ∆*phaC*, and ∆*phaZ* were 9.650 μg mL^−1^, 8.521 μg mL^−1^, and 10.543 μg mL^−1^, respectively. These findings indicate that the resistance of the mutant strains (∆*phaC*, ∆*phaZ*, ∆*phaC*/∆*phaZ*) to the HN-2 n-butanol extract was lower compared to the wild-type strain *PXO99^A^*, and the inhibitory effect of the HN-2 n-butanol extract on *Xoo* is superior to that of bacitracin. Taken together, these results suggest that *phaC/phaZ* is related to the drug resistance of *Xoo*, indicating that PHB plays a significant role in the drug resistance of *Xoo*. Additionally, these results demonstrate that the inhibitory effect of the HN-2 n-butanol extract on *Xoo* is more effective than that of commercial bacitracin.

### 2.3. Deletion of phaZ Reduced the Resistance of Xoo to the HN-2 n-Butanol Extract During Infection

**Figure 3:**
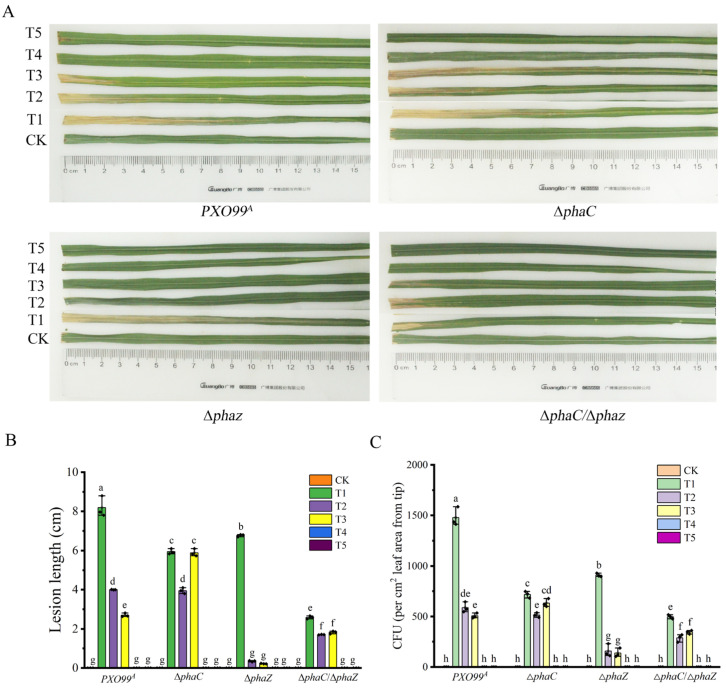
Effect of HN-2 n-butanol extract on the virulence of *Xoo* strains during rice infection. The lesion morphology on rice variety IR24 was observed 14 days post inoculation (dpi) with wild-type *PXO99^A^*, ∆*phaC*, ∆*phaZ*, and ∆*phaC*/∆*phaZ* (**A**). The measurements of lesion lengths were shown in sequence in (**B**). Additionally, bacterial colony-forming units (CFUs) used to quantify the multiplication of *Xoo* in rice leaves were presented in sequence in (**C**). Treatment conditions were as follows: CK (control): ddH_2_O; T1: *Xoo* inoculation without treatment; T2: treated with HN-2 n-butanol extract at one day post-*Xoo* inoculation; T3: treated with bacitracin at one day post-*Xoo* inoculation; T4: treated with HN-2 n-butanol extract one day before *Xoo* inoculation; and T5: treated with bacitracin one day before *Xoo* inoculation. The data are shown as the means with SD (±SD) with two-way ANOVA followed by Tukey’s multiple mean comparisons test method, the letters represent significance, *p* < 0.05.

The pathogenicity of *PXO99^A^* and its mutants (∆*phaC*, ∆*phaZ,* and ∆*phaC*/∆*phaZ*) to rice was evaluated using the leaf-cutting method. As shown in Figure 3, the lesion lengths for the wild-type *PXO99^A^*, ∆*phaC*, ∆*phaZ*, and ∆*phaC*/∆*phaZ* were 8.19 ± 0.54 cm, 5.94 ± 0.14 cm, 6.77 ± 0.06 cm, and 2.59 ± 0.08 cm, respectively (T1 in Figure 3A,B). The lesion lengths of the three mutants were significantly shorter than that of the wild-type strain *PXO99^A^*, with the ∆*phaC*/∆*phaZ* strain showing the weakest pathogenicity among all tested strains. Additionally, the bacterial multiplication in the inoculated leaves was assessed by counting the colonies grown on media. Compared with *PXO99^A^*, the population size of ∆*phaC* and ∆*phaZ* in rice was reduced approximately 51.42% and 38.50%, and 66.37% for ∆*phaC*/∆*phaZ* (T1 in Figure 3C). These results suggested that *phaC* and *phaZ* are critical for maintaining the pathogenicity of *Xoo*, likely by balancing intracellular energy metabolism through the regulation of PHB synthesis during plant infection.

Since the deletion of *phaC*/*phaZ* reduced *Xoo* drug resistance and the MIC_50_ to HN-2 n-butanol extract in vitro, we hypothesized that HN-2 n-butanol extract might also affect bacterial virulence and propagation of *Xoo* in rice during infection. To test this, we treated rice cultivar IR24 with HN-2 n-butanol extract one day before/after inoculation. The results showed that the preventive effect of HN-2 n-butanol extract and bacitracin on the leaves treated one day before *Xoo* inoculation was better than the therapeutic effect on the lesion leaves treated one day after *Xoo* inoculation (Figure 3). Moreover, compared to untreated *Xoo*-inoculated leaves (T1 in Figure 3), the treatment of spraying HN-2 n-butanol extract (T4 in Figure 3) and bacitracin (T5 in Figure 3) one day before inoculation had a 100% preventive effect. Additionally, both HN-2 n-butanol extract and bacitracin showed strong therapeutic effects on *Xoo*-infected rice plants (T2 and T3 in Figure 3), particularly on ∆*phaZ* mutants. The inhibition rates of HN-2 n-butanol extract and bacitracin on ∆*phaZ* were 94.83% and 96.75%, respectively, significantly outperforming the inhibition rates on wild-type strain *PXO99^A^* (51.16% and 67.03%, respectively). These results indicated the deletion of the *phaC*/*phaZ* gene not only reduced drug resistance of *Xoo* to the HN-2 n-butanol extract but also weakened its pathogenicity and reproductive capacity in rice, highlighting the crucial role of PHB synthesis in maintaining bacterial pathogenicity and drug resistance in *Xoo*. Furthermore, the HN-2 n-butanol extract exhibited superior inhibitory effects in controlling *Xoo* infection compared to the commercial antibiotic bacitracin, with particularly pronounced inhibition of the ∆*phaZ* mutant.

### 2.4. Mutation of phaC/phaZ Accelerated Cells Damage in Xoo Caused by the HN-2 n-Butanol Extract

**Figure 4:**
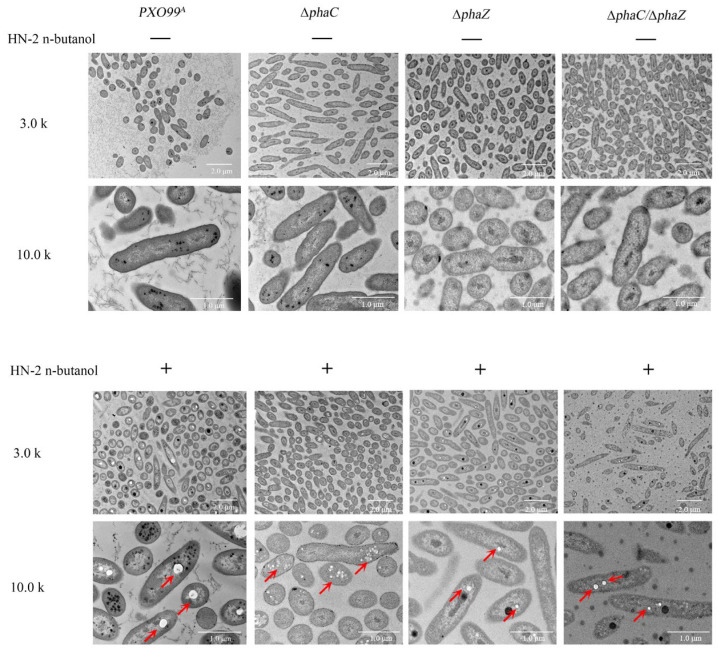
Morphology of *Xoo* cells under HN-2 n-butanol extract treatment. The morphology of *PXO99^A^* and ∆*phaC*, ∆*phaZ,* and ∆*phaC*/∆*phaZ,* cells was observed using transmission electron microscopy (TEM). The effects of HN-2 n-butanol extract on *Xoo* morphology were also assessed. Red arrows indicate the presence of PHB.

In our previous study, we investigated the morphological and ultrastructural changes in *Xoo* cells exposed to C15 surfactin A, finding that the cell walls became severely disrupted [32]. In this study, the deletion of *phaC*/*phaZ* reduced both the drug resistance to HN-2 n-butanol extract and the pathogenicity of *Xoo*. However, what specific morphological changes may occur as a result? To test this, we observed the morphological structures of strains (*PXO99^A^*, ∆*phaC*, ∆*phaZ*, ∆*phaC*/∆*phaZ*) treated with HN-2 n-butanol extract for 24 h using transmission electron microscope (TEM). The results were shown in Figure 4. Wild-type *PXO99^A^* cells without HN-2 n-butanol extract treatment were rod-shaped and well-formed, with ribosomes evenly distributed inside the cells. In contrast, ∆*phaC* cells appeared shorter and smaller, with ribosomes tending to aggregate near the cell wall, while ribosomes in ∆*phaZ* and ∆*phaC*/∆*phaZ* cells were more uniformly dispersed (Figure 4). After 24 h of treatment with HN-2 n-butanol extract, significant changes were observed in the cells of all strains (Figure 4). Wild-type *PXO99^A^* cells exhibited ribosome aggregation, with the formation of small, regular bright spots. In ∆*phaC* cells, these bright spots were smaller, irregular, and more numerous, with a concentrated distribution. In ∆*phaZ* and ∆*phaC*/*phaZ* cells, the bright spots were fewer and smaller compared to those in the wild-type *PXO99^A^*. Given these results, under treatment with HN-2 n-butanol extract, the ∆*phaC*, ∆*phaZ*, and ∆*phaC*/∆*phaZ* strains exhibited severe bacterial cell disruption, including membrane lysis, plasmolysis, and efflux of cytoplasmic components. This effect may be due to the role of PHB synthesis in maintaining cell wall stability. These results suggest that the HN-2 n-butanol extract accelerated cellular damage and had a more pronounced lethal effect on the mutant strains.

### 2.5. Loss of phaC/phaZ Increases Cell Membrane Sensitivity to the HN-2 n-Butanol Extract in Xoo

**Figure 5:**
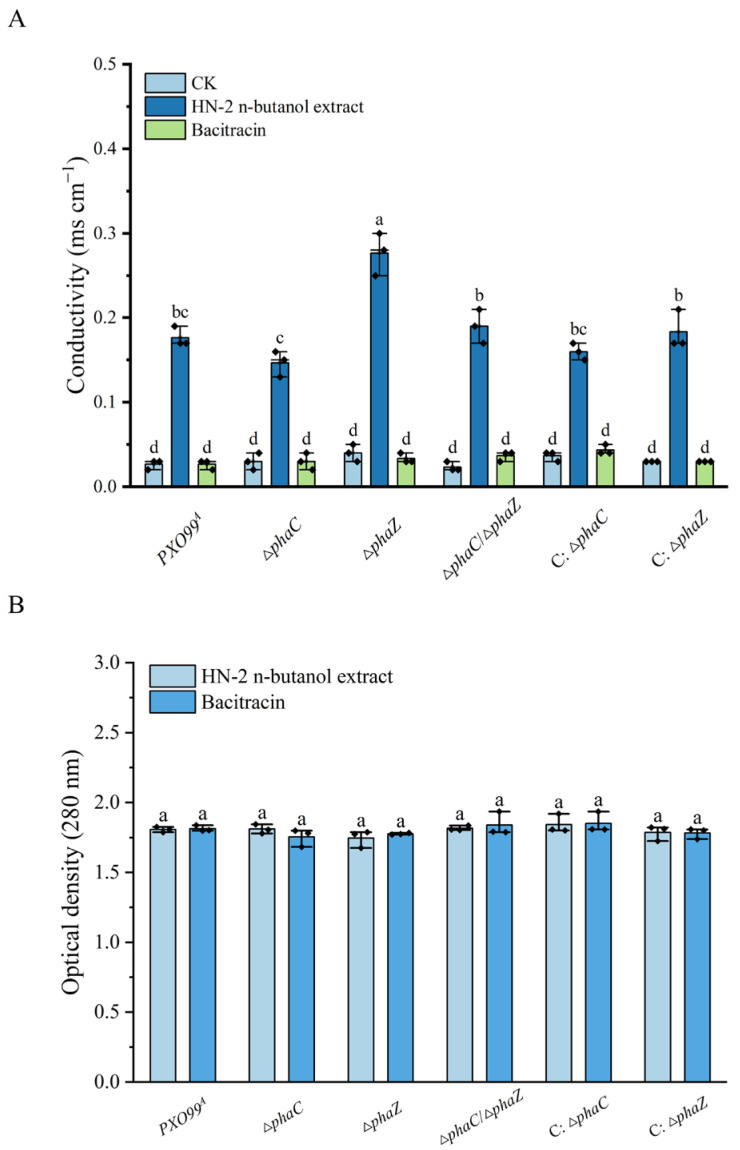
Influence of HN-2 n-butanol extract on the leakage of intracellular components in *Xoo*. The relative conductivity (**A**) and in vitro protein content (**B**) of the wild-type strain *PXO99^A^*, ∆*phaC*, ∆*phaZ*, ∆*phaC*/∆*phaZ*, C: ∆*phaC*, and C: ∆*phaZ* were measured under HN-2 n-butanol extract treatment, with bacitracin used as a control. The data are shown as the means with SD (±SD) with two-way ANOVA followed by Tukey’s multiple mean comparisons test method, the letters represent significance, *p* < 0.05.

In our previous report, we demonstrated that HN-2 n-butanol extract can cause damage to the cell wall of *PXO99^A^*, leading to the release of intracellular contents and disrupting cellular physiological functions [32]. We hypothesized that the loss of *phaC*/*phaZ* may increase the sensitivity of *Xoo* cell membranes to HN-2 n-butanol extract. To test this, the changes in conductivity of *Xoo* under HN-2 n-butanol extract treatment were measured. The results showed that the conductivity of ∆*phaZ* changed most significantly, which was about 0.30 ms cm^−1^ while the wild-type strain *PXO99^A^* and ∆*phaC*/∆*phaZ* was about 0.19 ms cm^−1^. The conductivity of the mutant ∆*phaC* had the lowest change of about 0.15 ms cm^−1^. Interestingly, bacitracin as a positive control had no significant effect on the conductivity of these strains, and there was no significant difference compared with the untreated group (Figure 5A). This observation was consistent with the results shown in Figure 2, where the inhibitory effect of the HN-2 n-butanol extract on the ∆*phaZ* was superior to that of bacitracin, further suggesting the potential of the HN-2 n-butanol extract as an effective agent for controlling *Xoo*-induced diseases.

To further assess the changes in cell membrane integrity, we examined the protein leakage in *Xoo* cells following treatment with HN-2 n-butanol extract and bacitracin. The degree of cell membrane damage was determined by measuring the optical density at 280 nm (OD_280_), reflecting the extent of protein leakage. The results, presented in Figure 5B, indicated that there was no significant difference in protein leakage between the *Xoo* strains after treatment with either HN-2 n-butanol extract or bacitracin. Taken together, the results indicated that the loss of *phaZ* may compromise the cell membrane integrity of *Xoo* under HN-2 n-butanol extract treatment, leading to leakage of intracellular electrolyte into the extracellular environment and an increase in solution conductivity, a phenomenon not observed in the ∆*phaC* strain compared to *PXO99^A^*. Therefore, the HN-2 n-butanol extract could serve as a promising treatment for *Xoo*-induced plant diseases.

### 2.6. Mutation of phaC and phaZ Increased Biofilm Formation in Xoo

**Figure 6:**
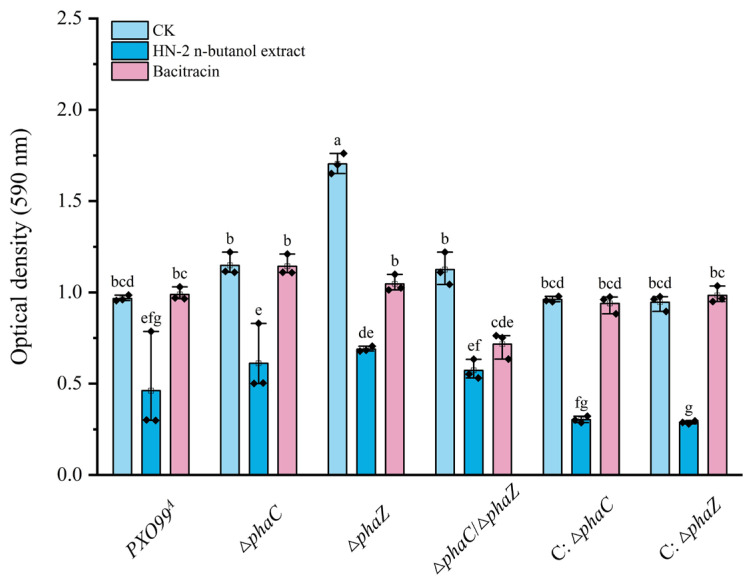
Determination of biofilm biosynthesis *Xoo* under HN-2 n-butanol extract treatment. The biofilm biosynthetic content of the wild-type strain *PXO99^A^*, ∆*phaC*, ∆*phaZ*, ∆*phaC*/∆*phaZ*, C: ∆*phaC*, and C: ∆*phaZ* was assessed by measuring the optical density (OD) at 570 nm to evaluate the effect of HN-2 n-butanol extract on *Xoo* biofilm biosynthesis. The data are shown as the means with SD (±SD) with two-way ANOVA followed by Tukey’s multiple mean comparisons test method, the letters represent significance, *p* < 0.05.

To further understand the mechanism underlying the effect of HN-2 n-butanol extract and bacitracin on the pathogenicity of *Xoo*, as well as the impact of PHA-related gene mutations, the biofilm formation of different strains (*PXO99^A^*, ∆*phaC*, ∆*phaZ*, ∆*phaC*/∆*phaZ*, C: ∆*phaC*, C: ∆*phaZ*) was assessed. Interestingly, after the mutation of *phaC* and *phaZ*, the biofilm formation increased compared to the wild-type *PXO99^A^* (Figure 6). This rise in biofilm production may be related to the stress response of *Xoo*, potentially indicating a compensatory mechanism to counteract the loss of PHB synthesis/degradation or to protect the cells under environmental or drug-induced stress. After treatment with HN-2 n-butanol extract, biofilm formation in wild-type *PXO99^A^* and ∆*phaC*, ∆*phaZ,* and ∆*phaC*/∆*phaZ* was significantly inhibited, with OD_590_ values reduced by 68.68%, 54.83%, 59.34%, and 49.72%, respectively, compared to the control group (Figure 6). HN-2 n-butanol extract appears to target biofilm formation in *Xoo*, and since biofilms serve as an essential protective barrier for bacterial cells, their disruption likely plays a key role in the inhibitory effect of HN-2 n-butanol extract on *Xoo* growth (Figure 2). The inhibition of biofilm formation might contribute to the reduced pathogenicity observed in infecting rice plants (Figure 3), as weakened biofilms diminish the defense mechanisms of *Xoo*, thereby enhancing the HN-2 n-butanol extract’s efficacy in *Xoo*-induced plant disease control.

**Figure 7:**
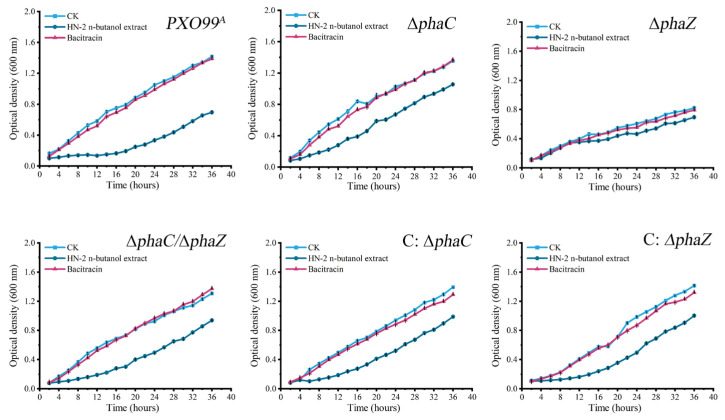
Determination of the growth rate of *Xoo* under HN-2 n-butanol extract treatment. The impact of HN-2 n-butanol extract on the growth rate of the wild-type strain *PXO99^A^*, ∆*phaC*, ∆*phaZ*, ∆*phaC*/∆*phaZ*, C: ∆*phaC*, and C: ∆*phaZ* was also determined by measuring the optical density (OD) at 600 nm. The data are shown as the means with SD (±SD).

The effects of HN-2 n-butanol extract and bacitracin on the growth curves of *Xoo* strains were also examined. As shown in (Figure 7), the growth rate of ∆*phaZ* strain was significantly lower than that of the wild-type *PXO99^A^*. Treatment with bacitracin did not cause significant changes in the growth curves of any strain compared to the control group. However, under the treatment of HN-2 n-butanol extract, the growth rate of all strains was markedly reduced. These findings suggest that HN-2 n-butanol extract exerts a stronger inhibitory effect on the growth of *Xoo*, particularly on strains with mutations in *phaC* and *phaZ*, further highlighting its potential as an effective agent for controlling *Xoo* infections. The decreased growth rate of ∆*phaZ* suggested that *phaZ* may play a critical role in *Xoo* growth, especially under stress conditions induced by the HN-2 n-butanol extract.

## 3. Discussion

Rice is a crucial staple crop for human consumption, but its growth is frequently compromised by plant pathogens, such as *Xanthomonas oryzae* pv. *oryzae* (*Xoo*), which causes bacterial leaf blight (BLB), one of the most common and devastating bacterial diseases in rice [34,35,36]. Chemical bactericides like bismerthiazol, zinc thiazole, and thiodiazole copper are often employed to control BLB. However, over-reliance on chemical control methods has led to the emergence of drug-resistant pathogens and raised concerns over environmental safety [6,7,37]. In light of this, biological control strategies offer a more sustainable and eco-friendlier alternative. *Bacillus* species, in particular, are known to produce a wide array of biologically active secondary metabolites that can inhibit plant pathogens and harmful rhizospheric microorganisms [9,38,39]. In this study, the antimicrobial activity of the fermentation broth extract from *Bacillus velezensis* HN-2 and its potentially controlling effect on *Xoo* were investigated to gain insights into bacterial drug resistance mechanisms. During this process, the resistance of *Xoo* to treatment agents may be associated with PHB production. Previous studies have reported that PHB could stabilize cell membranes by plugging small gaps caused by plasmolysis in hypertonic environments. Our findings align with previous studies demonstrating that PHB plays a role in bacterial resistance to stress conditions. For instance, Obruca et al. reported that PHB granules protect bacterial cells against osmotic imbalances, which supports our observation that PHB accumulation in the ∆*phaZ* mutant increased its resistance [28,40,41]. Also, our study extends these findings by showing that PHB also contributes to *Xoo* resistance against antimicrobial compounds such as HN-2 n-butanol extract, an aspect not previously reported. Similarly, studies by Sedlacek et al. showed that PHB protects bacterial cell integrity, consistent with our observation that ∆*phaZ* mutants exhibit higher resilience due to PHB accumulation [42].

Genome-wide analysis revealed that genes involved in polyhydroxyalkanoates (PHAs) metabolism were organized into gene clusters within *PXO99^A^* genomes. Specifically, five genes have been identified that play key roles in this metabolic pathway, including the gene encoding acetoacetyl-CoA reductase (*AACoAR*, *PXO_00406*), the PHA synthesis repressor (*phaR*, *PXO_00407*) gene, the poly (R)-hydroxyalkanoic acid synthase subunit phaC (*phaC*, *PXO_04210*) gene, the PHA synthase subunit (*phaE*, *PXO_04212*) gene, and poly-3-hydroxybutyrate (PHB) depolymerase (*phaZ*, *PXO_01811*) gene [29,33,43]. These key genes involved in PHB synthesis enable the production of PHB using various carbon sources, allowing organisms to store large amounts of energy and cope with environmental stresses. Knockout of *phaC* revealed PHB levels were significantly reduced in ∆*phaC*, while PHB levels in the ∆*phaZ* strains were accumulated compared to those in *PXO99^A^* (Figure 1B), suggesting *phaC* plays a key role in PHB biosynthesis in *PXO99^A^* and *phaZ* balanced the PHB content in vivo through depoly-PHB [44]. Notably, PHB production in *Xoo* was significantly induced by HN-2 n-butanol extract, further indicating that HN-2 n-butanol extract may stimulate PHB biosynthesis via activating compensatory pathways. This observation contrasts with findings that PHB biosynthesis is typically repressed under antimicrobial stress in some bacterial species [45,46]. The antibacterial activity assays in our study showed that HN-2 n-butanol extract could effectively inhibit the ∆*phaZ* and ∆*phaC* growth in petri dishes and decrease the MIC_50_ values (Table 1), indicating that HN-2 n-butanol extract may enhance the sensitivity of *Xoo* to its own treatment by inducing the production of large amounts of PHB (Figure 2). Within the PHB-related drug resistance pathways of *PXO99^A^*, *phaZ* plays a more significant role compared to *phaC* [47]. Interestingly, even after the mutation of *phaC*, the HN-2 n-butanol extract could still induce the production of amounts of PHB, suggesting that alternative PHB biosynthesis pathways may exist in *PXO99^A^* that collectively regulate PHB-related drug resistance pathways. Meanwhile, deletion of *phaC*/*phaZ* decreased the pathogenicity of *Xoo* in rice. The pathogenicity of ∆*phaC* and ∆*phaZ* strains was significantly lower than that of *PXO99^A^*, as was the multiplication, with the ∆*phaC*/∆*phaZ* strain showing an even more dramatic reduction (Figure 3). This suggests that *phaC*/*phaZ* play a crucial role in *PXO99^A^* virulence. The higher sensitivity of ∆*phaZ* to the HN-2 n-butanol extract further supports the notion that PHB hydrolysis is more important in the *PXO99^A^* drug resistance [30,44,48].

To further elucidate the relationship between PHB and drug resistance in *PXO99^A^*, transmission electron microscopy (TEM) was employed to observe the morphological characteristics of the bacteria. *PXO99^A^* without HN-2 n-butanol extract treatment exhibited rod-shaped and well-formed cells, with ribosomes evenly distributed throughout the cytoplasm, while ∆*phaC* appeared shorter and smaller, with ribosomes tending to aggregate near the cell wall, and ribosomes in ∆*phaZ* and ∆*phaC*/∆*phaZ* were more uniformly dispersed (Figure 4) [44]. This suggests that mutations in *phaC* and *phaZ* disrupt PHB metabolism, leading to alterations in *PXO99^A^* cell morphology, ribosomal distribution, and membrane stability. These findings are consistent with studies by Shen et al. [49], which demonstrated that PHB-deficient bacterial mutants often exhibit increased membrane permeability and structural instability. This may also explain the increased sensitivity to HN-2 n-butanol extract observed after the mutation of *phaZ*. Significant changes were observed in the cells of all strains after 24 h of treatment with HN-2 n-butanol extract (Figure 4). *PXO99^A^* exhibited ribosome aggregation, with the formation of small, regular bright spots, while in ∆*phaC*, these bright spots were smaller, irregular, and more numerous, with a concentrated distribution. In ∆*phaZ* and ∆*phaC*/∆*phaZ* cells, the bright spots were fewer and smaller compared to those in the *PXO99^A^*; these spots resembled previously observed PHB inclusions [28,45,50], which are known to contribute to cytomembrane repair (Figure 4). The size and number of these blank spots varied among strains, with wild-type *PXO99^A^* displaying the largest single spot, ∆*phaC* showing more but smaller spots, and ∆*phaZ* exhibiting the smallest and fewest spots. These observations are consistent with our hypothesis that PHB hydrolysis is critical for cell membrane repair and resistance to treatment agents. We hypothesize that PHB may play a role in drug resistance linked to its ability to repair cytomembranes [45,46].

Ion leakage and macromolecular protein leakage assays were performed to evaluate membrane integrity under treatment with HN-2 n-butanol extract (Figure 5). The significant increase in ion leakage in ∆phaZ mutants after HN-2 n-butanol extract treatment suggests that PHB contributes to membrane stabilization (Figure 5). However, protein leakage was insignificant [49]. This supports findings by Martínez-Tobón et al., who reported that PHB plays a role in preventing the uncontrolled efflux of cytoplasmic ions under stress conditions [51]. These results, combined with the antibacterial activity assays and transmission electron microscopy findings, suggest that HN-2 n-butanol extract induces micropore formation in the cell membrane, allowing ion leakage but not significant protein loss. The role of PHB in repairing these micropores may be limited, as indicated by the increased conductivity in the ∆*phaZ* strain. Biofilm formation is an important factor in bacterial pathogenicity [49,51]. The ∆*phaZ* strain demonstrated the strongest biofilm-forming ability (Figure 6), which contrasts with its lower pathogenicity. This may be due to PHB accumulation in the absence of PHA depolymerase activity, although the precise mechanism remains unclear. In terms of growth rate, the ∆*phaZ* strain also exhibited a significant reduction compared to the *PXO99^A^* (Figure 7), likely due to the inability to hydrolyze and utilize accumulated PHB, which in turn impaired bacterial growth. The double mutant ∆*phaC*/∆*phaZ* showed resistance and growth rates comparable to those of the *PXO99^A^*; this is consistent with the results of the antibacterial activity assays [51,52].

Taken together, these findings highlight that while PHB metabolism contributes to antimicrobial resistance in *Xoo*, it also plays a key role in bacterial virulence and structural integrity. The results extend previous research by demonstrating the dual role of PHB in stress adaptation and pathogenicity, suggesting that targeting PHB metabolism could serve as an effective strategy for controlling BLB. Further studies should focus on the regulatory mechanisms governing PHB metabolism and its interactions with other resistance pathways to develop novel control strategies against *Xoo*.

## 4. Materials and Methods

### 4.1. Plasmids, Bacteria and Culture Conditions

*Bacillus velezensis* HN-2, wild-type *Xanthomonas oryzae* pv. *oryzae* (*Xoo*) strain *PXO99^A^*, and its derived mutant strains (∆*phaC*, ∆*phaZ*, ∆*phaC*/∆*phaZ,* C*:* ∆*phaC*, C*:* ∆*phaZ*) were described in our previous studies [30,32]. The bacterial strains and plasmids used in this study are listed in Table S1. *Escherichia coli* DH5α and *Bacillus velezensis* HN-2 were grown in Luria-Bertani (LB) medium (10 g Tryptone, 5 g Yeast extract, 10 g NaCl per liter) at 37 °C with shaking at 180 rpm. *PXO99^A^* and its derived mutant strains (∆*phaC*, ∆*phaZ*, ∆*phaC*/*phaZ,* C*:* ∆*phaC*, C*:* ∆*phaZ*) were cultured in PSA medium (10 g Tryptone, 1 g monosodium glutamate, 10 g sucrose per liter) at 28 °C with shaking at 180 rpm. When cultured on solid medium plates, 15 g of agar per liter was added. Antibiotics were included as needed in both liquid and solid media.

### 4.2. Extraction of Fermentation Broth from Bacillus velezensis HN-2

The active substances were extracted from the fermentation broth of *Bacillus velezensis* HN-2 strain using the n-butanol extraction method described in our previous study [31,32]. The brief procedures are as follows: The fermentation broth was centrifuged at 8000 rpm and 25 °C to obtain the supernatant. The supernatant was then mixed with n-butanol in equal proportions and allowed to stand overnight at room temperature. Following this, the organic phase was separated using a separating funnel. The active substances in the organic phase were concentrated into a solid form by rotary evaporation at 60 °C. The solid residue was dissolved in a small amount of methanol and subsequently freeze-dried. The resulting extract was stored at −80 °C. Prior to use, the extract was dissolved in double distilled water (ddH_2_O) to a final concentration of 10 μg mL^−1^ and then filtered through a 0.22 µm syringe filter.

### 4.3. Antibacterial Activity Assays

The inhibitory effects of HN-2 n-butanol extract on the strain *PXO99^A^*, along with its derived mutants and complementary strains (∆*phaC*, ∆*phaZ*, ∆*phaC*/∆*phaZ,* C: ∆*phaC,* C*:* ∆*phaZ*), were assessed by the paper disk method [53,54]. The extract was dissolved in ddH_2_O to a final concentration of 10 μg mL^−1^ and subsequently filtered through a 0.22 µm syringe filter. A total of 10 μL of extract solution (10 μg mL^−1^) was applied to filter paper disks (6 mm in diameter, one per plate), which were then placed on the PSA medium plates pre-inoculated with the *Xoo* strains. A total of 100 μL bacterial suspension per plate, with an optical density at 600 nm, was 1.0 (OD_600_ = 1.0). Bacitracin was used as a control, and the experimental procedure was consistent with the treatment described above. The plates were incubated at 28 °C for 48 h, after which the diameter of the inhibition zones was measured.

### 4.4. Determination of 50% Minimum Inhibitory Concentration (MIC_50_)

The determination of the MIC_50_ against *Xoo* has been previously described in our earlier report [32]. Briefly, the HN-2 n-butanol extract and bacitracin were prepared at a concentration of 10 μg mL^−1^. The extract was then evenly mixed into PSA medium plates to achieve final concentrations of 2 μg mL^−1^, 4 μg mL^−1^, 6 μg mL^−1^, 8 μg mL^−1^, 10 μg mL^−1^. Similarly, bacitracin was mixed into PSA medium plates to obtain final concentrations of 5 μg mL^−1^, 10 μg mL^−1^, 15 μg mL^−1^, 20 μg mL^−1^, 25 μg mL^−1^. The bacterial suspensions (including *Xoo* wild-type *PXO99^A^* and its derived mutant strains ∆*phaC*, ∆*phaZ*, ∆*phaC*/∆*phaZ*) were adjusted to an optical density of OD_600_ = 0.1 and then serially diluted 50,000-fold using liquid PSA medium. A 100 μL aliquot of each bacterial suspension was evenly spread onto the PSA medium plates containing different concentrations of HN-2 n-butanol extract or bacitracin using a sterile cotton swab. Each bacterial strain was inoculated on separate plates. The plates were incubated at 28 °C for 72 h. Following incubation, the number of colonies was counted. The colony counts from PSA medium plates without the addition of HN-2 n-butanol extract or bacitracin served as controls.

### 4.5. Effects of HN-2 n-Butanol Extract on the Growth Curve of Xoo

The growth curve of *Xoo* strains was assayed to evaluate the effects of HN-2 n-butanol extract on *Xoo* growth [55]. The *Xoo* wild-type strain *PXO99^A^*, along with its derived mutant and complementary strains (∆*phaC*, ∆*phaZ*, ∆*phaC*/∆*phaZ,* C*:* ∆*phaC,* C*:* ∆*phaZ*), was cultured in PSA liquid medium to the logarithmic growth phase, approximately 24 h at 28 °C and 180 rpm. The bacterial cultures were then adjusted to an optical density of OD_600_ = 0.1, ensuring that the volume of each culture medium was consistent across samples. Subsequently, a specific volume of the HN-2 n-butanol extract (10 μg mL^−1^) or bacitracin solution (10 μg mL^−1^) was added to achieve a final concentration corresponding to 1 × MIC_50_ Control cultures, which received neither the extract nor bacitracin, were also set. All bacterial cultures were then incubated for 36 h at 28 °C and 180 rpm. The growth of each culture, indicated by the OD_600_ values, was measured every 2 h, and the value was recorded.

### 4.6. Measurement of Poly-3-Hydroxybutyrate (PHB) Content

The determination of PHB has been previously described in our earlier report [30]. The specific operational procedure is outlined below, with minor modifications. The *Xoo* wild-type strain *PXO99^A^*, along with ∆*phaC*, ∆*phaZ*, ∆*phaC*/∆*phaZ,* C: ∆*phaC,* and C: ∆*phaZ,* was cultured in PSA liquid medium at 28 °C and 180 rpm until an optical density of OD_600_ = 0.3 was reached. A specific amount of HN-2 n-butanol extract (10 μg mL^−1^) or bacitracin solution (10 μg mL^−1^) was then added to each bacterial culture to achieve a final concentration of 1 × MIC_50_. A control group without any extract or bacitracin was also set. The bacterial cultures were subsequently incubated for approximately 24 h at 28 °C and 180 rpm to reach the logarithmic growth phase. The bacterial cells were harvested by centrifugation at 8000 rpm and 4 °C for 10 min. The collected cells were stored overnight at −80 °C and then freeze-dried using a lyophilizer for 12 h to obtain dry bacterial powder.

The powdered bacteria were transferred into different glass test tubes independently. Chloroform and sodium hypochlorite (in a 1:1 ratio, with 2.5 mL of each) were added to the tubes, mixed thoroughly, and the tubes were sealed. The mixtures were shaken in an incubator at 28 °C and 180 rpm for 3 h. The mixtures were then transferred to new centrifuge tubes and centrifuged at 4000 rpm for 10 min. The upper layer was discarded, and chloroform in the lower layer was evaporated by heating in an oil bath at 100 °C, resulting in the formation of a poly-3-hydroxybutyrate (PHB) deposit. To dissolve the PHB deposit, 2.5 mL of concentrated sulfuric acid was added to the tubes, which were then sealed and heated in an oil-bath at 100 °C for 10 min. The tubes were subsequently cooled on ice, and then the solution was diluted 10-fold with ddH_2_O for ease of measurement. The optical density value at 235 nm (OD_235_) was measured to determine the PHB content. A standard curve for PHB was generated by weighing a known quantity of PHB and following the same procedure starting from the sulfuric acid addition step. The OD_235_ values were recorded and compared with the standard curve to quantify the PHB content in each experimental treatment.

### 4.7. Transmission Electron Microscopy

Transmission electron microscopy (TEM) was employed to assess the morphological changes induced by the HN-2 n-butanol extract on the *Xoo* wild-type strain *PXO99^A^* and its derived mutant strains (∆*phaC*, ∆*phaZ*, ∆*phaC*/∆*phaZ*) [30]. The *Xoo* strains were initially cultured in PSA liquid medium for 24 h at 28 °C and 180 rpm, after which the bacterial suspension was adjusted to an optical density of OD_600_ = 0.3. Subsequently, the HN-2 n-butanol extract or bacitracin solution was added to the cultures at a final concentration equivalent to the MIC_50_ for each strain, and the cultures were incubated for an additional 24 h under the same conditions. Following incubation, the bacterial cells were collected by centrifugation and fixed with 2.5% glutaraldehyde. The fixed samples were then sent for epon embedding, sectioning, and examination using a transmission electron microscope (Hitachi H-600, the Institute of Environment and Plant Protection, Chinese Academy of Tropical Agricultural Sciences).

### 4.8. Determination of Xoo Pathogenicity

The strains (*PXO99^A^*, ∆*phaC*, ∆*phaZ*, ∆*phaC*/∆*phaZ*) were cultured in PSA liquid medium for 24 h at 28 °C and 180 rpm until reaching the logarithmic growth phase. The bacterial suspension was then centrifuged for 2 min at 8000 rpm and room temperature. The supernatant was discarded, and the bacterial cells were retained. The cells were washed with ddH_2_O and resuspended in ddH_2_O, adjusting the optical density of each bacterial suspension to OD_600_ = 0.5. The method for inoculating rice with *Xoo* has been previously described in our earlier report [30]. Rice plants, including variety IR24 (with at least 12 plants), were inoculated at the tillering stage (five-leaves stage) using the leaf-clipping method. The inoculation was performed by cutting the rice leaves approximately 2–4 cm from the tip with clean scissors. At various time points, the HN-2 n-butanol extract and bacitracin solution (both at 10 μg mL^−1^) were diluted to the concentration corresponding to MIC_50_ and applied using 50 mL centrifuge tubes. The timing of the application was divided into two categories: one was spraying the solution one day before inoculation, and the other was spraying it one day after inoculation. Each experimental treatment involved inoculating ten rice leaves, with two leaves per plant. Rice plants that were inoculated with *Xoo* but not treated with the extract or bacitracin solution served as controls. Observation and photographs were conducted every 2 days post-treatment, and the lesion length was measured after 14 days.

### 4.9. Effects on Biofilm Formation

The ability of biofilm formation was assessed using a method adapted from Bae et al. [56,57]. The specific procedures are as follows: bacterial solutions *PXO99^A^*, ∆*phaC*, ∆*phaZ*, ∆*phaC*/∆*phaZ,* C: ∆*phaC, and* C: ∆*phaZ* were cultured at 28 °C and 180 rpm to an optical density of OD_600_ = 0.5. One milliliter of each bacterial solution was then transferred to a 24-well cell culture plate and incubated at 28 °C for 3 days. Before incubation, HN-2 n-butanol extract or bacitracin solution had been added to the wells at a final concentration corresponding to the MIC_50_ for each strain. Wells without HN-2 n-butanol extract or bacitracin served as controls. After incubation, the bacterial solution was discarded, and the wells were gently washed with ddH_2_O. Subsequently, 1 mL of 1% crystal violet was added to each well for 30 min for staining, followed by two washes with ddH_2_O. Once the plate was air-dried, 1 mL of anhydrous methanol was added to each well, and the plate was shaken at 70 rpm for 30 min at room temperature to dissolve the crystal violet. The absorbance of the resulting solution was then measured at OD_590_ to quantify the biofilm content.

### 4.10. Conductivity Measurement

The conductivity measurement of *Xoo* was performed using a method adapted from Li et al. [58] with minor modifications. Briefly, the bacteria to be tested (*PXO99^A^*, ∆*phaC*, ∆*phaZ*, ∆*phaC*/∆*phaZ, and* C: ∆*phaC,* C: ∆*phaZ*) were cultured in PSA liquid medium at 28 °C and 180 rpm until reaching the logarithmic growth phase. The bacterial solutions were then centrifuged at 5000 rpm for 2 min, washed with ddH_2_O, and this process was repeated three times. The bacteria were then resuspended in 20 mL ddH_2_O to achieve OD_600_ = 0.3. HN-2 n-butanol extracts or bacitracin solutions were added to the bacterial suspensions, and the mixtures were incubated for 2 h at a final concentration corresponding to the MIC_50_ for each strain. Centrifuge tubes containing only ddH_2_O served as a blank control. After 2 h, the conductivity of each bacterial suspension was measured using a conductivity meter (DDS-307, Leici, Shanghai, China).

### 4.11. Determination of Protein Leakage

The determination of protein leakage under HN-2 n-butanol extract treatment has been previously described [59]. The bacteria to be tested (*PXO99^A^*, ∆*phaC*, ∆*phaZ*, ∆*phaC*/∆*phaZ,* C: ∆*phaC,* C: ∆*phaZ*) were cultured in PSA liquid medium at 28 °C and 180 rpm until reaching the logarithmic growth phase. The cultures were then centrifuged at 10,000 rpm for 10 min; the bacteria were washed with PBS (2 mM KH_2_PO_4_, 8 mM Na_2_HPO_4_, 136 mM NaCl, 2.6 mM KCl, pH = 7.2) three times. Each bacterial pellet was resuspended in PBS to an OD_420_ = 0.8 HN-2 n-butanol extract or bacitracin solution was added to the bacterial suspensions, and mixtures were incubated for 2 h at a final concentration corresponding to MIC_50_ for each strain. Centrifuge tubes containing only ddH_2_O served as blank controls. After incubation, the values of OD_280_ were measured to determine protein leakage.

### 4.12. Statistical Analyses

Statistical analysis was performed using GraphPad Prism 9.0.0 (GraphPad Software, La Jolla, CA, USA) and OriginPro 2024b (OriginLab, Northampton, MA, USA). A two-way ANOVA followed by Tukey’s multiple comparisons test was performed. Statistical significance was defined as *p* ≤ 0.05. Each assay was conducted in three independent replicates to ensure the reliability of the results.

## 5. Conclusions

In conclusion, this study highlights the crucial role of PHB in drug-resistance of *PXO99^A^* and pathogenicity on rice. PHB likely contributes to membrane repair following damage, while its hydrolysis may provide energy to sustain the bacteria under stress. The *phaC* and *phaZ* genes appear to be key players in this process, though compensatory pathways may be activated when PHB metabolism is disrupted. Furthermore, our findings suggest that the HN-2 n-butanol extract can induce PHB biosynthesis, causing damage in the cell membrane. This damage may trigger PHB biosynthesis as a defense mechanism. Future research should focus on elucidating the specific mechanisms involved and confirming the role of surfactin in inducing PHB biosynthesis. These insights provide a deeper understanding of the molecular mechanisms underlying the drug-resistance of *PXO99^A^* to bactericides and pave the way for the development of novel, eco-friendly strategies for controlling bacterial leaf blight in rice.

## Figures and Tables

**Table 1:** MIC_50_ of HN-2 n-butanol extract and bacitracin for *Xoo* strains.

Strains	MIC_50_(HN-2 n-Butanol Extract) (μg/mL)	95% Confidence Interval (μg/mL)	Bacitracin MIC_50_ (μg/mL)	95% Confidence Interval (μg/mL)
*PXO99^A^*	0.450 a	0.363–0.660	9.650 b	9.128–10.208
∆*phaC*	0.282 c	0.22–0.337	8.521 d	7.716–9.289
C: ∆*phaC*	0.398 b	0.376–0.422	9.515 b	9.095–10.114
∆*phaZ*	0.213 d	0.201–0.224	10.543 a	8.896–12.144
C: ∆*phaZ*	0.384 b	0.371–0.403	9.487 b	9.014–10.076
∆*phaC/*∆*phaZ*	0.374 b	0.346–0.401	8.781 c	7.042–10.363

The data are shown as the means with two-way ANOVA followed by Tukey’s multiple mean comparisons test method, the letters represent significance, *p* < 0.05.

## Data Availability

The data presented in this study are available on request from the corresponding author.

## Supplementary Materials

The supporting information can be downloaded at: https://www.mdpi.com/article/10.3390/ijms26041601/s1.
