# Supplementary material for: Investigating Polyhydroxyalkanoate Synthesis for Insights into Drug Resistance in Xanthomonas oryzae pv. oryzae
Source: Int J Mol Sci. 2025 Feb 13;26(4):1601. doi: 10.3390/ijms26041601 (PMC11855620; doi:10.3390/ijms26041601)
Supplement: Supplementary file 1 [file ijms-26-01601-s001.zip › ijms-3450190-supplementary.pdf]

Table S1. Strains and plasmids used in this study.

Strains and Plasmids	Relevant Genotype or Characteristics	Source
Escherichia coli DH5α	Hosts and vector donors for plasmid construction	Transgen Bio Inc
Xanthomonas. oryzae pv. *oryzae* *PXO99^{A}*	wild-type strain	Laboratory collection
Δ*phaC*	*phaC* gene mutant strain	Laboratory collection
Δ*phaZ*	*phaZ* gene mutant strain	Laboratory collection
Δ*phaC*/Δ*phaZ*	*phaC* and *phaZ* gene mutant strain	Laboratory collection
C: Δ*phaC*	*phaC* gene complementary strain	Laboratory collection
C: Δ*phaZ*	*phaZ* gene complementary strain	Laboratory collection
Bacillus velezensis HN-2	wild-type strain	Laboratory collection
